# S5-1 Physical literacy in different European contexts: research-based knowledge for practical applications

**DOI:** 10.1093/eurpub/ckad133.023

**Published:** 2023-09-11

**Authors:** Chair(s):, Amika Singh, Johannes Carl

**Affiliations:** Mulier Institute, The Netherlands; Department Sportwissenschaft und Sport, Friedrich-Alexander-Universität

## Abstract

The term and concept of physical literacy is increasing in popularity in both policy and practice and is increasingly applied in the fields such as sports, public health and education.

Physical literacy is the ability to move with competence and confidence in a variety of physical activities. Although there is widespread consensus that physical literacy has the potential to act as the basis for a lifelong active life, there is little known about how it is assessed, operationlized, and applied.

The aim of this symposium is to describe what is currently happening in the European context. Therefore, in this symposium we will bring together researchers and practitioners to discuss the asseessment, operationlization and application of the physical literacy concept across four European countries.

Presentation 1) Process of developing a physical literacy consensus statement for England (UK)

Presentation 2) Towards national monitoring of physical literacy in Denmark

Presentation 3) Application of the concept of physical literacy in the context of water: aquatic physical literacy (Belgium)

Presentation 4) Developing an individual-based advice for sports participation and physical activity in children using the concept of Physical Literacy (the Netherlands).

The discussant of the symposium who will lead the discussion after the individual presentations will ensure exchange of knowledge, expertise and experiences among audience and presenters.

